# Mental health outcomes of developmental coordination disorder in late adolescence

**DOI:** 10.1111/dmcn.13469

**Published:** 2017-05-16

**Authors:** Ian Harrowell, Linda Hollén, Raghu Lingam, Alan Emond

**Affiliations:** ^1^ Centre for Child and Adolescent Health School of Social and Community Medicine University of Bristol Bristol UK; ^2^ Institute of Health and Society University of Newcastle Newcastle UK

## Abstract

**Aim:**

To assess the relationship between developmental coordination disorder (DCD) and mental health outcomes in late adolescence.

**Method:**

Data were analyzed from the Avon Longitudinal Study of Parents and Children. Moderate‐to‐severe DCD was defined at 7 to 8 years according to the DSM‐IV‐TR criteria. Mental health was assessed at 16 to 18 years using self‐reported questionnaires: Strengths and Difficulties Questionnaire, Short Moods and Feelings Questionnaire, and the Warwick–Edinburgh Mental Well‐being Scale. Logistic and linear regressions assessed the associations between DCD and mental health, using multiple imputation to account for missing data. Adjustments were made for socio‐economic status, IQ, and social communication difficulties.

**Results:**

Adolescents with DCD (*n*=168) had an increased risk of mental health difficulties (total Strengths and Difficulties Questionnaire score) than their peers (*n*=3750) (odds ratio 1.78, 95% confidence interval 1.12–2.83, adjusted for socio‐economic status and IQ). This was, in part, mediated through poor social communication skills. Adolescent females with DCD (*n*=59) were more prone to mental health difficulties than males. Greater mental well‐being was associated with better self‐esteem (*β* 0.82, *p*<0.001).

**Interpretation:**

Individuals with DCD, particularly females, had increased risk of mental health difficulties in late adolescence. Interventions that aim to promote resilience in DCD should involve improving social communication skills and self‐esteem.

## What this paper adds

- Mental health difficulties in developmental coordination disorder persist into late adolescence.
- Adolescent females with developmental coordination disorder may have greater risk of mental health difficulties.
- Social communication difficulties mediate the relationship between developmental coordination disorder and mental health.

This article is commented on by Wagner on pages 891–892 of this issue.

This article's abstract has been translated into Spanish and Portuguese.

Follow the links from the abstract to view the translations.

## Abstract (es)

**Objetivo:**

Evaluar la relación entre el trastorno del desarrollo de la coordinación (TDC) y parámetros de salud mental en la adolescencia tardía.

**Método:**

Se analizaron datos del *Estudio Longitudinal Avon de Padres e Hijos*. TDC moderado a severo se definió a los 7 a 8 años según los criterios del DSM‐IV‐TR. La salud mental se evaluó a los 16‐18 años mediante cuestionarios autoadministrados: SDQ (Cuestionario de Fortalezas y Dificultades), SMFQ (Cuestionario Corto de Emociones y Estado de Ánimo) y Escala de bienestar mental de Warwick‐Edimburgh. La asociación entre TDC y parámetros de salud mental fue evaluada por medio de regresiones logísticas y lineales. Se utilizó la imputación múltiple para dar cuenta de los datos faltantes. Se hicieron ajustes en base a situación socioeconómica, coeficiente intelectual y dificultades de comunicación social.

**Resultados:**

Los adolescentes con TDC (n = 168) tuvieron un mayor riesgo de dificultades de salud mental (puntuación total del Cuestionario de Fortalezas y Dificultades) que sus pares (n = 3750) (odds ratio 1,78, intervalo de confianza del 95%: 1,12‐2,83; ajustado por situación económica y coeficiente intelectual). Esto fue, en parte, mediado por dificultades en la comunicación social. Las mujeres adolescentes con TDC (n = 59) fueron más propensas a tener dificultades de salud mental que los hombres con TDC. Mayor bienestar mental se asoció con una mayor autoestima [*β* 0,82, p <0,001].

**Interpretación:**

Las personas con TDC, en particular las mujeres, tuvieron un mayor riesgo de dificultades de salud mental en la adolescencia tardía. Las intervenciones que tienen por objeto promover la resiliencia en la TDC deberían implicar el mejoramiento de las habilidades de comunicación social y de la autoestima.

## Resumo

**Objetivo:**

Avaliar a relação entre o Transtorno do Desenvolvimento da Coordenação (TDC) e resultados de saúde mental no final da adolescência.

**Método:**

Os dados foram analisados a partir do Estudo Longitudinal Avon de Pais e Crianças. O TDC moderado a grave foi definido entre 7 e 8 anos de acordo com os critérios do DSM‐IV‐TR. A saúde mental foi avaliada dos 16 aos 18 anos usando questionários autoaplicáveis: Questionário de Força e Dificuldades (SDQ), Questionário Curto sobre Humor e Sentimentos (SMQF) e Escala de Bem‐Estar Mental de Warwick‐Edinburgh. Regressões logísticas e lineares avaliaram as associações entre TDC e saúde mental, usando a imputação múltipla para dar conta de dados faltantes. Ajustes foram feitos para o status socioeconômico, QI, e dificuldades de comunicação social.

**Resultados:**

Os adolescentes com TDC (n = 168) tiveram um risco aumentado de dificuldades de saúde mental (pontuação total do Questionário de Força e Dificuldades) do que os seus pares (n = 3750) (odds ratio 1,78, intervalo de confiança de 95% 1,12‐2,83, ajustado para status econômico e QI). Isso foi, em parte, mediado por pobres habilidades de comunicação social. Adolescentes do sexo feminino com TDC (n = 59) foram mais propensas a problemas de saúde mental do que os homens. Maior bem‐estar mental foi associado com melhor autoestima [*β* 0,82, p <0,001].

**Interpretação:**

Indivíduos com TDC, particularmente mulheres, tiveram maior risco de dificuldades em saúde mental no final da adolescência. As intervenções que visam promover a resiliência no TDC devem envolver o aprimoramento das habilidades de comunicação social e auto‐estima.

**Abbreviations**
ALSPAC: Avon Longitudinal Study of Parents and Children
DCD: Developmental coordination disorder
SMFQ: Short Moods and Feelings Questionnaire
SDQ: Strengths and Difficulties Questionnaire
WEMWBS: Warwick–Edinburgh Mental Well‐being Scale

Developmental coordination disorder (DCD) is a common neurodevelopmental condition which affects 2% to 6% of the population.^1^ It is characterized by deficits in motor coordination that interfere with a child's ability to perform everyday tasks and academic activities. Previous research has shown that children with DCD have an increased risk of mental health difficulties compared with their peers,^2^ and findings from cross‐sectional studies suggest that young adults with DCD have a higher risk of experiencing internalizing problems, particularly mood impairments.^3^ The few longitudinal studies that have been conducted have demonstrated greater prevalence of psychosocial problems for those with DCD in the long term, although recruitment from clinical samples, where risk of comorbidity is higher, and lack of strict diagnostic criteria limit the strength of the conclusions that can be drawn.^4^

The mechanisms for DCD leading to poor mental health outcomes are not well understood. A monozygotic twin study reported that twins with DCD demonstrated higher levels of depressive symptoms than their co‐twins without DCD, which suggests that mental health problems in DCD may be explained by unique environmental experiences.^5^ The current study tests the Environmental Stress Hypothesis,^6^ which considers poor motor coordination to be the primary stressor, which leads to multiple secondary stressors, such as frustration at school, negative peer interactions, or bullying,^7^ which, in turn, contribute to poor self‐esteem and subsequently internalizing problems.^8^ Conversely, protective factors, such as social support,^9^ guard against this cascade. Over time, the cumulative experience of positive and negative factors determines overall mental health and well‐being.

Previous work on the Avon Longitudinal Study of Parents and Children (ALSPAC) cohort has shown that poor mental health outcomes at 9 to 10 years in children with DCD were associated with poor social communication skills, increased bullying, and poor peer relationships.^2^ In this follow‐up study using the ALSPAC cohort, we have re‐examined the risk of mental health difficulties at 16 to 18 years in those with diagnosed DCD at 7 years of age, and hypothesized that those with DCD would continue to have a greater risk of mental health problems than their peers. We have also assessed the positive outcome of overall mental well‐being, which we hypothesized would be lower in those with DCD. Finally, we aimed to assess the impact of poor social communication skills, low self‐esteem, peer relationships, and bullying on mental health and well‐being in adolescents with DCD.

## Method

### ALSPAC

ALSPAC is a large population‐based birth cohort that invited all pregnant females in the Avon area, south‐west England, with expected dates of delivery between 1 April 1991 and 31 December 1992 to take part.^10^ The original sample comprised 14 062 live‐born children. ALSPAC has collected data on a large range of socio‐economic, environmental, and health measures for parents and children, using questionnaires, face‐to‐face assessments, and linked health/education data. The study website contains details of all the data that are available through a fully searchable data dictionary (http://www.bris.ac.uk/alspac/researchers/data-access/data-dictionary/). Ethical approval was obtained from the ALSPAC Ethics and Law Committee and the local research ethics committees.

### Measures

#### Exposure: moderate‐to‐severe DCD

Children were defined as having DCD according to the adaption of the DSM‐IV‐TR criteria set out in the Leeds Consensus Statement 2006.^11^ Children were defined as having DCD at 7 to 8 years of age if they met all four of the DSM‐IV‐TR criteria: (1) poor motor coordination; (2) functional limitation in activities of daily living or academic achievement; (3) absence of another neurological/visual disorder; and (4) absence of severe learning difficulty (IQ<70). Definition of children with DCD in this cohort has been described in detail previously.^1^

Motor skills were assessed in a research clinic between 7 and 8 years of age using the ALSPAC coordination test, which consisted of three subtests of the Movement Assessment Battery for Children.^12^ The subtests were chosen to represent the three domains of coordination: balance (heel‐to‐toe walking), manual dexterity (placing pegs task), and ball skills (throwing bean bag into box). Age‐adjusted ALSPAC coordination scores for each subtest were generated and summed to give an overall score.^1^ Functional limitations were assessed using results from the English National Curriculum writing test at 5 to 7 years of age and a parent‐reported activities of daily living scale. Those with known visual, developmental or neurological conditions, or with an IQ<70 were excluded from case definition of DCD.

A cohort of 6902 children had all the data required for full assessment. Those with a motor score below the fifteenth centile and who failed their National Curriculum writing test, or scored less than the fifteenth centile in the activities of daily living scale, were defined as having moderate‐to‐severe DCD.

#### Outcomes

##### Strength and Difficulties Questionnaire

The Strengths and Difficulties Questionnaire (SDQ) was self‐reported at 16 years 6 months. The SDQ is a standardized questionnaire widely used to screen for mental health difficulties in young people and comprises 20 items relating to four different psychosocial scales (five items each): emotional symptoms; conduct; hyperactivity/inattention; and peer problems. Responses were scored using a three‐point Likert scale, and answers summed to give a ‘total difficulties’ score out of 40. In a large survey of UK children, those with a score in the worst decile have been shown to have a 15‐fold increase in the odds of having an independently diagnosed psychiatric disorder.^13^ Scores were dichotomized using this 10% cut‐off to define those with a high risk of mental health difficulties.

##### Short Mood and Feelings Questionnaire

The Short Mood and Feelings Questionnaire (SMFQ) was self‐reported at 17 years 6 months. The SMFQ is a 13‐point validated questionnaire used to identify depressive symptoms. It has good validity and reliability in determining the presence of depression when a young person scores 11 or more.^14^ We dichotomized the scores using this cut‐off to define those with high risk of depression.

##### The Warwick–Edinburgh Mental Well‐being Scale

The Warwick–Edinburgh Mental Well‐being Scale (WEMWBS) questionnaire was self‐reported at 17 years 6 months. The WEMWBS comprises 14 positively worded items and responses were scored using a five‐point Likert scale. Responses were summed to provide a single score out of 70, which indicates overall mental well‐being. WEMWBS score demonstrated good validity and reliability in adolescent populations in the UK.^15^ It does not have a defined cut‐off for normality and so was analyzed as a continuous variable.

#### Confounding variables

Potential confounding variables were selected based on previous literature and our own univariate analyses against both the exposure (DCD) and the outcome measures.

Child‐related variables assessed were sex, gestation, and birthweight. In this follow‐up cohort, sex was associated with both exposure and outcomes.

Parental mental health and socio‐economic status were associated with both DCD and child mental health in previous analyses.^2^ Presence of maternal depression was taken from a self‐report questionnaire at 12 years 6 months. Socio‐economic status was assessed using the ALSPAC Family Adversity Index. The Family Adversity Index is derived from responses to a questionnaire about childhood adversity and socio‐economic status, which mothers completed during pregnancy. The index comprises 18 items assigned a score of 1 if adversity is present and 0 if it is absent, giving a total possible score of 18.

#### Mediating variables

The following variables were considered as potential mediators, based on previous literature: IQ;^2^ hyperactivity/impulsivity;^2^ friendship support and bullying;^7^ self‐esteem;^8^ and social communication skills.^9^

##### IQ

IQ was measured using the short version of the Wechsler Intelligence Scale for Children III, applied in a research clinic at 8 years 6 months of age. IQ shows high stability over time when measured in school‐age children onwards.^16^

##### Hyperactivity/impulsivity

Difficulties with hyperactivity are incorporated in the total SDQ score as one of the subscales, and so were only adjusted for in the SMFQ analyses, using those in the worst decile of the SDQ hyperactivity/inattention subscale to denote significant hyperactivity.

##### Social communication

Social communication difficulties were measured using the Social and Communications Disorders Checklist,^17^ completed by the main caregiver at 16 years 6 months. It comprises 12 items relating to the individual's social communication ability and cognition, each with three responses (not true/quite or sometimes true/very or often true). Answers are summed to give a score between 0 and 36; a score of 9 or above was used to identify social communication difficulty trait.^18^

##### Self‐esteem

Self‐esteem was assessed at 17 years 6 months using the Bachman revision of the Rosenberg's Self‐esteem Scale, which comprises 10 statements relating to self‐esteem, each with four responses (strongly disagree/disagree/agree/strongly agree), which are summed to give a total score.^19^

##### Friendship support

Friendship support was assessed at 13 years 6 months using five questions from the Cambridge Hormones and Moods Project Friendship Questionnaire;^20^ a higher score indicated less supportive peer relationships.

##### Bullying

Bullying was assessed at 15 to 16 years as part of a self‐reported questionnaire. Respondents were asked how often they were upset by name‐calling/exclusion or bullying in the last year, with four possible responses (never/rarely/sometimes/most days). Children were classified as being overt victims of bullying if they answered ‘sometimes’ or ‘most days’.

### Analyses

Logistic regressions were used to assess the associations between DCD and mental health difficulties on the SDQ and SMFQ. Multivariable models were created to adjust for the effect of the confounding and mediating variables sequentially. A ‘variable‐focused model’ of resilience was used to consider how different variables influenced the risk of psychopathology in children with DCD compared with controls.^21^ Model 1 adjusted for child and parental factors. Model 2 adjusted for IQ. Model 3 adjusted for social communication difficulties and hyperactivity. Pearson goodness of fit *χ* ^2^ tests were used to ensure satisfactory model fit. Linear regressions were used to examine the association between mental well‐being on the WEMWBS and each of the covariates. Tests were two‐tailed, with an a priori set alpha‐level of *p*<0.05.

Multiple imputation using chained equations was used to impute missing data in the covariates only. Analysis of imputed data sets helps to minimize attrition bias and improve precision of estimates.^22^ Logistic regressions were used to determine which variables strongly predicted missingness; these were included in the imputation models (Appendix S1 and Table SI, online supporting information). Analyses were performed using all available data (Tables SII–SIV, online supporting information) and imputed data sets for the cohort as a whole, and then stratified by sex. Results from analyses of the imputed data sets, where the effect of attrition is minimized, are presented. All analyses were performed using Stata version 13.1 (StatCorp, Jacksonville, FL, USA).

## Results

### Sample

For those assessed for DCD at 7 to 8 years of age (*n*=6902), data for the SDQ at 16 years 6 months were available for 3918 children (168 [4.3%] with DCD), whereas data for the SMFQ and WEMWBS at 17 years 6 months were available for 3177 children (130 [4.1%] with DCD). Compared with those who were followed‐up, those with missing outcome data were more likely to be male, have lower IQ, have lower socio‐economic status, and have mothers who reported depression (Appendix S1). Characteristics of the children with DCD and controls who responded to the SDQ are compared in Table 1. In this follow‐up cohort, those with DCD were more likely to have poor social communication skills, lower self‐esteem, less supportive friendships, and report more frequent bullying.

**Table 1:** Comparison of the characteristics of the adolescents with and without developmental coordination disorder (DCD) for whom outcome data (Strengths and Difficulties Questionnaire) were available

	Control (max. *n*=3750)	DCD (max. *n*=168)	Effect size for test	*p*
Male sex	1780 (48)	109 (65)	OR 2.04, 95% CI 1.48–2.82	**<0.001** **a**
Gestation ≥37wks	3570 (95)	159 (95)	OR 0.89, 95% CI 0.45–1.77	0.742^a^
Birth weight ≥2500g	3552 (96) (*n*=3706)	155 (93) (*n*=166)	OR 0.61, 95% CI 0.32–1.15	0.123^a^
Maternal depression on self‐report at 12.5y – yes	689 (21) (*n*=3350)	37 (26) (*n*=143)	OR 1.34, 95% CI 0.92–1.98	0.126^a^
Median (25th/75th centiles) FAI	0 (0–1)	1 (0–2)	*r*=0.03	0.074^c^
Mean (SE) IQ at 8y	107.7 (0.26) (*n*=3424)	96.8 (1.42) (*n*=142)	*d*=0.71	**<0.001** **b**
SCDC at 16.5y (score >8)	394 (11) (*n*=3603)	35 (21) (*n*=163)	OR 2.22, 95% CI 1.51–3.28	**<0.001** a
Median (25th/75th centiles) self‐esteem score at 17.5y	29 (24–33) (*n*=2428)	27 (21–31) (*n*=100)	*r*=0.06	**0.002** **c**
Median (25th/75th centiles) friendship score at 13.5y	2 (1–3) (*n*=2676)	3 (2–4) (*n*=110)	*r*=0.07	**<0.001** **c**
Bullying frequency at 15.5y				
Sometimes/most days	410 (15) (*n*=2810)	29 (26) (*n*=110)	OR 2.10, 95% CI 1.35–3.24	**0.001** a
Most days only	60 (2) (*n*=2810)	9 (8) (*n*=110)	OR 4.10, 95% CI 1.97–8.46	**<0.001** a

Data are reported as *n* (%) unless otherwise indicated. Bold denotes statistical significance. ^a^Logistic regression. ^b^Student's *t*‐test. ^c^Mann–Whitney *U* test. FAI, Family Adversity Index; SE, standard error; SCDC, Social Communication Difficulties Checklist; *r*, effect size for Mann–Whitney *U* test; *d*, Cohen's d statistic of effect size for Student's *t*‐test.

#### SDQ

The odds ratios (OR) and 95% confidence intervals (CI) of mental health difficulties measured by the SDQ using the imputed data sets are presented in Table 2.

**Table 2:** Mental health difficulties measured using the Strengths and Difficulties Questionnaire (SDQ) and Short Mood and Feelings Questionnaire (SMFQ) at 16 to 18 years in adolescents with developmental coordination disorder (DCD) compared to controls, using multiple imputation data

	Unadjusted OR (95% CI)	*p*	Model 1 OR (95% CI)^a^	*p*	Model 2 OR (95% CI)^b^	*p*	Model 3 OR (95% CI)^c^	*p*
SDQ total difficulties score, with a 10th centile cut‐off (*n*=3918)	2.15 (1.38–3.35)	**0.001**	2.09 (1.32–3.29)	**0.002**	1.78 (1.12–2.83)	**0.024**	1.32 (0.77–2.26)	0.321
SDQ hyperactivity subscale (*n*=3950)	2.12 (1.41–3.21)	**<0.001**	1.84 (1.20–2.82)	**0.005**	1.53 (1.00–2.37)	**0.052**	1.28 (0.80–2.04)	0.312
SDQ emotional subscale (*n*=3942)	1.36 (0.81–2.27)	0.247	1.55 (0.91–2.64)	0.102	1.47 (0.86–2.51)	0.149	1.19 (0.68–2.09)	0.532
SDQ peer problems subscale (*n*=3940)	2.87 (1.88–4.40)	**<0.001**	2.64 (1.71–4.07)	**<0.001**	2.24 (1.56–3.76)	**<0.001**	2.14 (1.36–3.37)	**0.001**
SDQ conduct subscale (*n*=3944)	1.22 (0.65–2.28)	0.528	1.18 (0.62–2.23)	0.614	1.02 (0.54–1.94)	0.950	0.61 (0.30–1.25)	0.170
SMFQ, with a cut‐off of 11 (*n*=3177)	1.51 (1.00–2.28)	**0.048**	1.56 (1.03–2.38)	**0.042**	1.40 (0.91–2.15)	0.124	1.26 (0.81–1.96)	0.303

Bold denotes statistical significance. ^a^Model 1 adjusted for sex, maternal depression, and Family Adversity Index. ^b^Model 2 – Model 1 plus IQ. ^c^Model 3 – Model 2 plus difficulties in social communication (note: hyperactivity was also adjusted for in SMFQ analysis). OR, odds ratio; CI, confidence interval.

Cross‐tabulation for the total difficulties SDQ score showed that 14.9% (*n*=25) of those with DCD were at risk of mental health difficulties versus 7.5% (*n*=282) of those without DCD. After adjusting for socio‐economic factors (Model 1) and IQ (Model 2), the odds of difficulty were attenuated but remained high. However, further adjustment for co‐existing social communication difficulties (Model 3) substantially attenuated the relationship. Using the SDQ subscales, children with DCD were more likely to have problems with hyperactivity and peer relationships, and these associations were similarly attenuated in the fully adjusted model.

When stratified by sex (Table 3), males with DCD were no more likely than same‐sex controls to have mental health difficulties, as measured by the total score. However, they were more likely to report hyperactivity/impulsivity and peer relationship difficulties. The association with hyperactivity attenuated after adjustment but the association with peer difficulties persisted after adjusting for socio‐economic factors, IQ, and social communication difficulties. In comparison, females with DCD had increased odds of a mental health difficulty on a number of measures when compared with same‐sex controls. They had greater odds of difficulty on the total SDQ and with hyperactivity/impulsivity, emotions, and peer relationships. All associations attenuated after adjustment for social communication skills.

**Table 3:** Mental health difficulties measured using the Strengths and Difficulties Questionnaire (SDQ) and Short Mood and Feelings Questionnaire (SMFQ) in males and females with developmental coordination disorder (DCD), compared to same‐sex controls, using multiple imputation data

		Unadjusted OR (95% CI)	*p*	Model 1 OR (95% CI)^a^	*p*	Model 2 OR (95% CI)^b^	*p*	Model 3 OR (95% CI)^c^	*p*
Males	SDQ total difficulties score, with a 10th centile cut‐off (*n*=1889)	1.61 (0.86–3.01)	0.136	1.44 (0.76–2.72)	0.267	1.24 (0.65–2.38)	0.510	0.96 (0.45–2.05)	0.923
	SDQ hyperactivity subscale (*n*=1911)	1.72 (1.05–2.83)	**0.032**	1.64 (0.99–2.72)	0.056	1.32 (0.79–2.22)	0.295	1.19 (0.68–2.09)	0.541
	SDQ emotional subscale (*n*=1905)	1.05 (0.42–2.65)	0.921	0.92 (0.36–2.33)	0.853	0.90 (0.35–2.32)	0.826	0.81 (0.31–2.12)	0.662
	SDQ peer problems subscale (*n*=1904)	2.87 (1.71–4.81)	**<0.001**	2.70 (1.60–4.56)	**0.001**	2.54 (1.50–4.33)	**0.001**	2.40 (1.38–4.16)	**0.002**
	SDQ conduct subscale (*n*=1903)	0.72 (0.30–2.00)	0.533	0.64 (0.23–1.78)	0.389	0.53 (0.19–1.52)	0.244	0.35 (0.11–1.06)	0.064
	SMFQ, with a cut‐off of 11 (*n*=1374)	1.48 (0.82–2.66)	0.188	1.39 (0.77–2.52)	0.270	1.22 (0.67–2.23)	0.511	1.11 (0.60–2.05)	0.736
Females	SDQ total difficulties score, with a 10th centile cut‐off (*n*=2029)	3.31 (1.75–6.26)	**<0.001**	3.42 (1.77–6.62)	**0.001**	2.91 (1.49–5.69)	**0.002**	2.01 (0.90–4.48)	0.089
	SDQ hyperactivity subscale (*n*=2039)	2.52 (1.21–5.24)	**0.013**	2.58 (1.20–5.51)	**0.015**	2.27 (1.05–4.91)	**0.036**	1.53 (0.65–3.62)	0.328
	SDQ emotional subscale (*n*=2037)	2.16 (1.13–4.14)	**0.020**	2.20 (1.13–4.27)	**0.020**	2.05 (1.05–4.00)	**0.036**	1.61 (0.79–3.26)	0.186
	SDQ peer problems subscale (*n*=2036)	2.54 (1.18–5.48)	**0.018**	2.52 (1.16–5.48)	**0.019**	2.24 (1.03–4.91)	**0.043**	1.70 (0.75–3.86)	0.204
	SDQ conduct subscale (*n*=2041)	2.19 (0.97–4.92)	0.059	2.15 (0.95–4.90)	0.068	1.90 (0.83–4.37)	0.130	0.97 (0.37–2.55)	0.954
	SMFQ, with a cut‐off of 11 (*n*=1803)	1.91 (1.05–3.45)	**0.033**	1.82 (0.99–3.34)	0.054	1.65 (0.89–3.06)	0.109	1.47 (0.78–2.77)	0.233

Bold denotes statistical significance. ^a^Model 1 adjusted for: maternal depression and Family Adversity Index. ^b^Model 2 – Model 1 plus IQ. ^c^Model 3 – Model 2 plus difficulties in social communication (note: hyperactivity was also adjusted for in SMFQ analysis). OR, odds ratio; CI, confidence interval.

#### SMFQ

The ORs and 95% CIs of mental health difficulties measured by the SMFQ using the imputed data sets are presented in Table 2.

A weak association was found between reported depressive symptoms on the SMFQ and DCD; 24.6% (*n*=32) of DCD cases reported symptoms versus 17.8% (*n*=541) of those without DCD. After adjusting for socio‐economic factors, this association persisted but was attenuated after adjustment for IQ and social communication skills.

When stratified by sex (Table 3), males were no more likely than same‐sex controls to report depressive symptoms. However, females were more likely to report depressive symptoms compared with same‐sex controls. This association attenuated after adjustment for social communication skills.

#### WEMWBS

The linear associations between well‐being and psychosocial factors for those with DCD using the imputed data sets are presented in Table 4. Males with DCD reported a higher mean WEMWBS score than females with DCD (49.2 vs 43.4, *t*=−3.64; *p*<0.001). Higher well‐being scores were positively associated with higher self‐esteem for both males and females with DCD. For females only, lower self‐esteem was associated with poor social communication skills.

**Table 4:** Linear associations between mental well‐being score on the Warwick–Edinburgh Mental Well‐being Scale (WEMWBS) and psychosocial factors for adolescents with developmental coordination disorder (DCD), using multiple imputation data and stratified by sex

Psychosocial factor	All (*n*=130)		Males (*n*=79)		Females (*n*=51)	
	Coefficient (SE)	*p*	Coefficient (SE)	*p*	Coefficient (SE)	*p*
FAI score^a^	−0.72 (0.49)	0.143	−0.77 (0.57)	0.183	−0.52 (0.81)	0.536
IQ	0.05 (0.04)	0.363	0.01 (0.06)	0.918	0.12 (0.08)	0.155
SCDC score^b^	−0.27 (0.19)	0.175	−0.10 (0.22)	0.660	−0.67 (0.33)	**0.050**
Self‐esteem score	0.82 (0.09)	**<0.001**	0.77 (0.13)	**<0.001**	0.77 (0.14)	**<0.001**
Friendship score^c^	−0.44 (0.40)	0.286	−0.81 (0.43)	0.065	0.08 (0.83)	0.923

Bold denotes statistical significance. ^a^Higher score indicates more family adversity. ^b^Higher score indicates increased difficulties. ^c^Higher score indicates less supportive friendships. SE, standard error; FAI, Family Adversity Index; SCDC, Social and Communication Difficulties Checklist.

## Discussion

The results of this prospective study using a large UK cohort showed that children with DCD had a greater risk of mental health difficulties in late adolescence than their peers. They had particular difficulties with hyperactivity and peer relations and were also more likely to report depressive symptoms than their peers. When compared with same‐sex controls, females with DCD were more likely to report emotional difficulties and depressive symptoms, whereas males with DCD experienced greater peer problems. These mental health difficulties were mediated by poor social communication skills. In those with DCD, higher mental well‐being was associated with higher self‐esteem. These results add robust longitudinal data, using a strict definition of DCD, to the growing body of evidence linking motor coordination problems with mental health problems in later life, and highlight the important contributory role of poor social communication skills.

The key strengths of this study are use of a large population‐based cohort, which avoids the bias inherent in clinical samples, and the prospective follow‐up over 10 years. We were able to adjust for multiple confounders and covariates and were also able to consider a positive outcome of well‐being, collected separately from the mental health difficulties.

The main limitation is missing data. ALSPAC, in common with all cohorts, suffers from considerable attrition, which we have attempted to account for by using multiple imputation, an increasingly recognized method of dealing with missing data to minimize bias.^22^ Previous work on this cohort found that children with DCD had greater risk of mental health difficulties on the SDQ (OR 4.23, 95% CI 3.10–5.77) and SMFQ (OR 2.08, 95% CI 1.36–3.19) when compared with their peers at 9 to 10 years.^2^ In late adolescence, the risk was not as severe or pervasive. This may represent a true reduction in risk of psychopathology with age, but it must be acknowledged that attrition may have contributed to this lower observed risk. The factors that predicted missingness in this sample are also known to be associated with DCD, and so it is possible that drop‐out of the more severe cases has led to an underestimation of risk.

Few studies have been large enough to assess sex differences in psychosocial outcomes in DCD. In our previous work on ALSPAC, no sex effect was found at 9 to 10 years.^2^ In contrast, at 17 years, we found that when compared to their same‐sex controls, females with DCD had a higher risk of self‐reported emotional and depressive problems than males with DCD. In their population‐based cohort study, Sigurdsson et al. found higher levels of maternally reported anxiety in adolescent males compared with females who had previous indicators of motor impairment in childhood.^4^ One study of 7‐ to 8‐year‐olds olds referred to an occupational therapy service reported that females with DCD were more likely to experience peer problems than males on the parent‐reported SDQ.^23^ Another study found motor skills to be a potentially important factor affecting psychosocial well‐being in adolescent females.^24^ Population‐based research has also demonstrated that females, in general, are at greater risk of reporting depressive symptomatology in adolescence.^14^ It may be that a higher risk of depression in adolescence combined with both direct and indirect consequences of DCD make females with the condition were more likely to develop mental health difficulties than their male counterparts. This is concerning because females with DCD are thought to be an underdiagnosed group.

The Environmental Stress Hypothesis proposes that the primary stressor of poor motor coordination leads to a series of secondary stressors over time, which, in turn, lead to poor mental health.^6^ Of particular interest is the impact of poor motor coordination on a child's social development. Children with DCD often struggle to perform in games and sports, and may avoid activities that highlight their impairments. Consequently, they may become socially isolated, and develop low self‐esteem, which we found in this cohort.^2^ Self‐perceptions are an important factor influencing the relationship between poor motor skills and emotional outcomes.^25^ It has also been proposed that poor motor skills in childhood may impair important executive functions, such as cognitive flexibility, which impact negatively on social understanding and therefore relationships.^26^ Our work on the ALSPAC cohort has consistently shown the mediating effect of social communication skills and self‐esteem on risk of mental health problems in DCD, relationships that are complex but have important clinical implications.

In light of mounting evidence that poor motor coordination and mental health are linked, clinicians need to manage not only the primary deficits in DCD, but also the secondary features that may influence the trajectory of mental health. The importance of social communication skills, self‐esteem and peer support in mediating psychosocial outcomes in DCD has been increasingly highlighted in numerous studies. Clinical, psychological, and social interventions targeted on these domains may represent the best opportunity to improve mental health and well‐being of children with DCD. This has been recognized in programmes such as the Partnering for Change initiative,^27^ which focuses on providing a more holistic approach to managing DCD in schools. The concept has also been investigated in a randomized trial, which found that motor and psychosocial skills improved in 4‐ to 6‐year‐olds who took part in a whole‐class physical activity programme called ‘Animal Fun’, which promoted social interaction.^28^ More intervention‐focused research is needed to determine the longitudinal benefits for adolescents and young adults with DCD, who are an important but less well understood group.

In summary, the work on the ALSPAC cohort has demonstrated longitudinally that poor mental health is an important consequence of DCD, especially so for females in adolescence. Factors that consistently affect these relationships are social communication skills, peer support, bullying, and self‐esteem.

### Conclusion

Clinicians need to be aware of and screen for mental health difficulties in young people with DCD. Combining therapy for motor coordination difficulties with interventions that help promote social interaction, self‐esteem, and positive peer relationships may improve mental health outcomes in these individuals.

## Supporting information


**Appendix S1**: Missing data and multiple imputation.
**Table SI**: Factors which predict missingness in the data (Strengths and Difficulties Questionnaire at 16y 6mo) for the whole cohort
**Table SII**: Mental health difficulties measured using the Strengths and Difficulties Questionnaire (SDQ) and the Short Moods and Feelings Questionnaire (SMFQ) at 16 to 18 years in adolescents with developmental coordination disorder (DCD) compared to controls, using all available data
**Table SIII**: Mental health difficulties measured using the Strengths and Difficulties Questionnaire (SDQ) and Short Mood and Feelings Questionnaire (SMFQ) in males and females with developmental coordination disorder (DCD), compared to same‐sex controls, using all available data
**Table SIV**: Linear associations between mental well‐being score on the Warwick–Edinburgh Mental Well‐being Scale (WEMWBS) and psychosocial factors for adolescents with developmental coordination disorder (DCD), using all available data and stratified by sex
