# Supplementary material for: Mental health outcomes of developmental coordination disorder in late adolescence
Source: Dev Med Child Neurol. 2017 May 16;59(9):973–9. doi: 10.1111/dmcn.13469 (PMC5573907; doi:10.1111/dmcn.13469)
Supplement: Supplementary file 1 — Appendix S1: Missing data and multiple imputation. Table SI: Factors which predict missingness in the data (Strengths and Difficulties Questionnaire at 16y 6mo) for the whole cohort Table SII: Mental health difficulties measured using the Strengths and Difficulties Questionnaire (SDQ) and the Short Moods and Feelings Questionnaire (SMFQ) at 16 to 18 years in adolescents with developmental coordination disorder (DCD) compared to controls, using all available data Table SIII: Mental health difficulties measured using the Strengths and Difficulties Questionnaire (SDQ) and Short Mood and Feelings Questionnaire (SMFQ) in males and females with developmental coordination disorder (DCD), compared to same‐sex controls, using all available data Table SIV: Linear associations between mental well‐being score on the Warwick–Edinburgh Mental Well‐being Scale (WEMWBS) and psychosocial factors for adolescents with developmental coordination disorder (DCD), using all available data and stratified by sex [file DMCN-59-973-s001.docx]

**Appendix S1**: Missing data and multiple imputation

Missing data are a problem in all cohort studies, particularly when data are collected over a long period and from different sources, such as in ALSPAC. Of those children assessed for DCD at 7 years, 3918 had data for the total SDQ score at 16 years 6 months and 3177 had data available for the MFQ and WEMWBS at 17 years 6 months.

The multi-variate models for logistic regression used nine different covariates. Taking the SDQ analysis, 1704 children (43%) had no missing values in any of the covariates, 1091 (28%) had a single missing value and 1123 (29%) had between 1 and 5 missing values.

Multiple imputation is a technique which can help improve validity of research with significant missing data by accounting for bias introduced by loss to follow-up (Sterne et al. 2009). In this study, multiple imputation using chained equations was performed using the “ICE” command in Stata v.13.1 (StatCorp Inc, USA). Twenty stacked datasets were generated and used in the final analysis which used the “MIM” command.

Variables used in the imputation model were all of those included in the final regression model and those which predicted missingness in the covariates. In ALSPAC, it is known that those from lower socio-economic groups are less likely to be followed up. Therefore, we used logistic regression to explore if the following factors were associated with missingness in our sample: ALSPAC Family Adversity Index (which incorporates a number of different socioeconomic factors), maternal age at delivery, antenatal depression and antenatal anxiety. We also assessed birthweight, gestation and IQ measured by the Wechsler Intelligence Scale for Children II at a mean age of 8.7 years.

The factors which strongly predicted missingness (p<0.05) were maternal age at delivery, antenatal depression, the Family Adversity Index and IQ; these were all included in the final imputation model. Binary and categorical variables used logistic, ordinal and multinomial as appropriate, specified in the imputation model. IQ was normally distributed and used linear regression.

By including extra variables which explain missingness in the imputation model, as well as the covariates of interest, the assumption of “missing at random” is supported.

| **Table SI**: Factors which predict missingness in the data (Strengths and Difficulties Questionnaire at 16 years 6 months) for the whole cohort. | | | |
| --- | --- | --- | --- |
|  | **Responders**  (max. n=3918) | **Non-responders**  (max. n=2728) | *p* |
| Gender – male | 2616 (48) n=3918 | 1467 (54) n=2728 | **<0.001^a^** |
| Gestation - >=37 weeks | 3729 (95) n=3918 | 2553 (94) n=2728 | **0.005 ^a^** |
| Birthweight - >=2500g | 3707 (96) n=3872 | 2557 (95) n=2557 | **0.020 ^a^** |
| IQ at 8 years - mean (standard error) | 107.3 (0.26) n=2566 | 101.6 (0.34) n=1887 | **<0.001^b^** |
| Family Adversity Index – median (25^th^/75^th^ centiles) | 0 (0-1) n=3918 | 1 (0-2) n=2728 | **<0.001^c^** |
| Maternal education – Degree attained | 761 (20) n=3859 | 233 (9) n=2539 | **<0.001 ^a^** |
| Maternal depression on self-report at 12.5 years - yes | 726 (21) n=3493 | 315 (26) n=1225 | **<0.001 ^a^** |
| Data are reported as *n* (%) unless otherwise indicated. Bold denotes statistical significance. ^a^Logistic regression. ^b^Student’s *t*-test. ^c^Mann–Whitney *U* test. | | | |

| **Table SII:** Mental health difficulties measured using the Strengths and Difficulties Questionnaire (SDQ) and the Short Moods and Feelings Questionnaire (SMFQ) at 16 to 18 years in adolescents with developmental coordination disorder (DCD) compared to controls, using all available data. | | | | | | | | | | | | |
| --- | --- | --- | --- | --- | --- | --- | --- | --- | --- | --- | --- | --- |
|  | **Unadjusted** OR (95% CI) | n | *p* | **Model 1** OR^a^ (95% CI) | n | *p* | **Model 2** OR^b^ (95% CI) | n | *p* | **Model 3** OR^c^ (95% CI) | n | *p* |
| SDQ Total Difficulties score, with a 10^th^ centile cut-off | 2.14 (1.38 - 3.34) | 3918 | **0.001** | 2.18 (1.34 - 3.55) | 3445 | **0.002** | 2.19 (1.31 - 3.66) | 3170 | **0.003** | 1.59 (0.85 - 2.94) | 3058 | 0.142 |
| SDQ hyperactivity subscale | 2.12 (1.41 - 3.20) | 3950 | **<0.001** | 2.17 (1.39 - 3.40) | 3472 | **0.001** | 2.02 (1.24 - 3.27) | 3192 | **0.004** | 1.69 (0.99 - 2.87) | 3078 | 0.063 |
| SDQ emotional subscale | 1.36 (0.81 - 2.27) | 3942 | 0.239 | 1.68 (0.95 - 2.97) | 3465 | 0.071 | 1.70 (0.91 - 3.15) | 3187 | 0.092 | 1.28 (0.67 - 2.46) | 3075 | 0.460 |
| SDQ peer problems subscale | 2.87 (1.88 - 4.40) | 3940 | **<0.001** | 2.68 (1.69 - 4.23) | 3464 | **<0.001** | 2.43 (1.47 - 4.02) | 3186 | **0.001** | 1.99 (1.16 - 3.38) | 3072 | **0.012** |
| SDQ conduct subscale | 1.22 (0.65 - 2.28) | 3944 | 0.527 | 1.41 (0.74 - 2.68) | 3468 | 0.292 | 1.51 (0.78 - 2.92) | 3189 | 0.208 | 0.88 (0.41 - 1.86) | 3075 | 0.743 |
| SMFQ, with a cut-off of 11 | 1.51 (1.00 - 2.28) | 3177 | **0.050** | 1.60 (0.99 - 2.60) | 2640 | 0.054 | 1.42 (0.85 - 2.38) | 2494 | 0.176 | 1.32 (0.75 - 2.34) | 2120 | 0.333 |
| Bold denotes statistical significance. ^a^Model 1 adjusted for sex, maternal depression, and Family Adversity Index. ^b^Model 2 – Model 1 plus IQ. ^c^Model 3 – Model 2 plus difficulties in social communication (note: hyperactivity was also adjusted for in SMFQ analysis). OR, odds ratio; CI, confidence interval. | | | | | | | | | | | | |

| **Table SIII:** Mental health difficulties measured using the Strengths and Difficulties Questionnaire (SDQ) and Short Mood and Feelings Questionnaire (SMFQ) in males and females with developmental coordination disorder (DCD), compared to same-sex controls, using all available data. | | | | | | | | | | | | | |
| --- | --- | --- | --- | --- | --- | --- | --- | --- | --- | --- | --- | --- | --- |
|  |  | **Unadjusted** OR (95% CI) | n | *p* | **Model 1** OR^a^ (95% CI) | n | *p* | **Model 2** OR^b^ (95% CI) | n | *p* | **Model 3** OR^c^  (95% CI) | n | *p* |
| **Boys** | SDQ total difficulties score, with a 10^th^ centile cut-off | 1.61 (0.86 - 3.01) | 1889 | 0.143 | 1.38 (0.69 - 2.78) | 1654 | 0.362 | 1.45 (0.71 - 2.98) | 1503 | 0.311 | 1.00 (0.42 - 2.39) | 1459 | 0.980 |
|  | SDQ hyperactivity | 1.72 (1.05 - 2.83) | 1911 | **0.031** | 1.97 (1.16 - 3.35) | 1674 | **0.012** | 1.70 (0.96 - 3.02) | 1518 | **0.070** | 1.49 (0.79 - 2.79) | 1472 | 0.210 |
|  | SDQ emotional | 1.04 (0.42 - 2.64) | 1905 | 0.924 | 0.69 (0.21 - 2.25) | 1668 | 0.535 | 0.80 (0.24 - 2.67) | 1514 | 0.719 | 0.62 (0.18 - 2.17) | 1470 | 0.464 |
|  | SDQ peer problems | 2.87 (1.71 - 4.81) | 1904 | **0.001** | 2.58 (1.47 - 4.54) | 1668 | **0.001** | 2.59 (1.42 - 4.72) | 1515 | **0.002** | 2.20 (1.16 - 4.19) | 1469 | **0.023** |
|  | SDQ conduct | 0.72 (0.26 - 2.01) | 1903 | 0.528 | 0.75 (0.26 - 2.11) | 1667 | 0.581 | 0.78 (0.27 - 2.25) | 1513 | 0.652 | 0.46 (0.15 - 1.49) | 1467 | 0.203 |
|  | SMFQ, with a cut-off of 11 | 1.48 (0.83 - 2.66) | 1374 | 0.187 | 1.32 (0.67 - 2.62) | 1163 | 0.423 | 1.02 (0.48 - 2.17) | 1102 | 0.958 | 0.93 (0.41 - 2.13) | 931 | 0.881 |
| **Girls** | SDQ total difficulties score, with a 10^th^ centile cut-off | 3.31 (1.75 - 6.26) | 2029 | **0.001** | 3.90 (1.94 - 7.83) | 1791 | **0.001** | 3.98 (1.87 - 8.49) | 1667 | **<0.001** | 3.00 (1.18 - 7.63) | 1599 | **0.022** |
|  | SDQ hyperactivity | 2.52 (1.21 - 5.24) | 2039 | **0.012** | 2.92 (1.30 - 6.56) | 1798 | **0.010** | 3.15 (1.30 - 7.63) | 1674 | **0.010** | 2.51 (0.94 - 6.72) | 1606 | 0.074 |
|  | SDQ emotional | 2.16 (1.13 - 4.14) | 2037 | **0.020** | 2.70 (1.36 - 5.34) | 1797 | **0.004** | 2.65 (1.24 - 5.66) | 1673 | **0.011** | 2.05 (0.92 - 4.58) | 1605 | 0.083 |
|  | SDQ peer problems | 2.54 (1.18 - 5.48) | 2036 | **0.023** | 2.75 (1.24 - 6.08) | 1796 | **0.011** | 2.09 (0.84 - 5.24) | 1671 | 0.114 | 1.59 (0.60 - 4.20) | 1603 | 0.349 |
|  | SDQ conduct | 2.19 (0.97 - 4.92) | 2041 | 0.064 | 2.55 (1.10 - 5.89) | 1801 | **0.028** | 2.93 (1.23 - 7.01) | 1676 | **0.021** | 1.59 (0.54 - 4.67) | 1608 | 0.401 |
|  | SMFQ, with a cut-off of 11 | 1.91 (1.05 - 3.45) | 1803 | **0.033** | 2.17 (1.07 - 4.40) | 1477 | **0.032** | 2.19 (1.04 - 4.61) | 1392 | **0.041** | 2.11 (0.92 - 4.83) | 1189 | 0.083 |
| Bold denotes statistical significance. ^a^Model 1 adjusted for: maternal depression and Family Adversity Index. ^b^Model 2 – Model 1 plus IQ. ^c^Model 3 – Model 2 plus difficulties in social communication (note: hyperactivity was also adjusted for in SMFQ analysis). OR, odds ratio; CI, confidence interval. | | | | | | | | | | | | | |

| **Table SIV:** Linear associations between mental well-being score on the Warwick-Edinburgh Mental Well-being Scale (WEMWBS) and psychosocial factors for adolescents with developmental coordination disorder (DCD), using all available data and stratified by gender. | | | | | | | | | |
| --- | --- | --- | --- | --- | --- | --- | --- | --- | --- |
|  | **All** | | | **Boys** | | | **Girls** | | |
| **Psychosocial Factor** | Coefficient (SE) | n | *p* | Coefficient (SE) | n | *p* | Coefficient (SE) | n | *p* |
| FAI score^a^ | -0.72 (0.49) | 130 | 0.142 | -0.77 (0.57) | 79 | 0.183 | -0.52 (0.81) | 51 | 0.529 |
| IQ | 0.05 (0.05) | 118 | 0.363 | 0.01 (0.07) | 71 | 0.913 | 0.11 (0.10) | 47 | 0.181 |
| Social and Communication Difficulties Checklist score^b^ | -0.23 (0.20) | 98 | 0.248 | -0.12 (0.22) | 64 | 0.628 | -0.64 (0.30) | 34 | **0.050** |
| Self-esteem score | 0.82 (0.09) | 130 | **<0.001** | 0.77 (0.13) | 79 | **<0.001** | 0.77 (0.14) | 51 | **<0.001** |
| Friendship score^c^ | -0.47 (0.37) | 110 | 0.214 | -0.80 (0.45) | 69 | 0.072 | 0.09 (0.81) | 41 | 0.922 |
| Bold denotes statistical significance. ^a^Higher score indicates more family adversity. ^b^Higher score indicates increased difficulties. ^c^Higher score indicates less supportive friendships. SE, standard error; FAI, Family Adversity Index; SCDC, Social and Communication Difficulties Checklist. | | | | | | | | | |
